# WHO, HOW, AND WHEN DOES PRIMARY CARE USE THE 4MS? CLINICIAN PERSPECTIVES OF THE AGE-FRIENDLY FRAMEWORK IN AN FQHC

**DOI:** 10.1093/geroni/igad104.1863

**Published:** 2023-12-21

**Authors:** Jenny Knecht-Fredo, Erica Husser, Donna Fick, Angela Hogue, Marie Mulvihill, Lisa Lorelli

**Affiliations:** Pennsylvania State University, State College, Pennsylvania, United States; Penn State University, State College, Pennsylvania, United States; Penn State University, State College, Pennsylvania, United States; Primary Health Network, Sharon, Pennsylvania, United States; The Primary Health Network, Lewistown, Pennsylvania, United States; The Primary Health Network, Beaver Falls, Pennsylvania, United States

## Abstract

Older adults are at higher risk of living with multiple chronic health conditions and confront health care systems ill-prepared to manage the underlying complexities of providing care to older people. The Geriatric Workforce Enhancement Program (GWEP) aims to transform primary care to improve the health of older adults. Our GWEP is a collaboration between the Penn State College of Nursing and Pennsylvania’s largest Federally Qualified Health Center (FQHC), the Primary Health Network (covering 16 counties [13 rural]). We aim to disseminate the 4Ms of an Age-Friendly Health System (What Matters, Medication, Mentation, and Mobility). Although AFHS is being disseminated across the nation, there is little research regarding clinicians’ perspectives of the 4Ms framework in primary care. After 3 years of program delivery, we administered a PHN system-wide survey to 614 employees with 307 returned (50% response rate) regarding 4Ms knowledge, clinicians’ responsibilities, timing of assessments, how results inform care, perceptions on the role of the electronic medical record (EMR), competence, barriers, and facilitators to providing Age-Friendly care. Analysis includes a subset of 40 registered nurses (n=21) and nurse practitioners/physician’s assistants/physicians (n=19). Results suggest the medical providers were perceived as most likely to assess What Matters and Medication and multiple team members assessed Mentation and Mobility. 65% either agree or strongly agree that the EMR should prompt them to review the 4Ms with their patients. Survey results provide understanding of the barriers, specific roles, workflows, and EMR alterations needed to implement AFHS care into the busy culture of primary care clinics.

